# Supplementary material for: Gender Differences among Healthcare Providers in the Promotion of Patient-, Person- and Family-Centered Care—And Its Implications for Providing Quality Healthcare
Source: Healthcare (Basel). 2023 Feb 14;11(4):565. doi: 10.3390/healthcare11040565 (PMC9957388; doi:10.3390/healthcare11040565)
Supplement: Supplementary file 1 [file healthcare-11-00565-s001.zip › healthcare-2166877-supplementary.pdf]

Supplemental Materials

Supplemental Table S1. Database search of keywords used to cross reference "patient-centered care" literature in six medical specialties.

Specialty	"OR" Terms	"AND" Terms
Obstetrics & Gynecology	obstetrics & gynecology, obstetric service, obstetrics, obstetric care, gynecologic care, gynecology, gynecologic service, obstetrician, gynecologist[1]	Patient-centered care, patient centered care, patient-centred care, patient centred care
Pediatrics	pediatrics, pediatric, pediatrician, pediatric care, pediatric service	
Dermatology	dermatology, dermatologists, dermatologic service	
Radiology	radiology, radiology service, radiologist, radiologists, diagnostic radiology, interventional radiology	
Neurosurgery	neurosurgery, neurosurgeon, neurosurgical procedure	
Orthopedic Surgery	orthopedic procedure, orthopedics, orthopaedic surgery, orthopaedics, orthopaedic surgeon	

[1]Both spellings of the word gynecology (gynecology vs gynaecology) were used in the search
